# Simple knockout by electroporation of engineered endonucleases into intact rat embryos

**DOI:** 10.1038/srep06382

**Published:** 2014-10-01

**Authors:** Takehito Kaneko, Tetsushi Sakuma, Takashi Yamamoto, Tomoji Mashimo

**Affiliations:** 1 Institute of Laboratory Animals, Graduate School of Medicine, Kyoto University, Kyoto 606-8501, Japan; 2 Department of Mathematical and Life Sciences, Graduate School of Science, Hiroshima University, Higashi-Hiroshima 739-8526, Japan

## Abstract

Engineered endonucleases, such as zinc-finger nucleases (ZFNs), transcription activator-like effector nucleases (TALENs), and the clustered regularly interspaced short palindromic repeat (CRISPR)/CRISPR-associated (Cas) system, provide a powerful approach for genome editing in animals. However, the microinjection of endonucleases into embryos requires a high skill level, is time consuming, and may cause damage to embryos. Here, we demonstrate that the electroporation of endonuclease mRNAs into intact embryos can induce editing at targeted loci and efficiently produce knockout rats. It is noteworthy that the electroporation of ZFNs resulted in an embryonic survival rate (91%) and a genome-editing rate (73%) that were more than 2-fold higher than the corresponding rates from conventional microinjection. Electroporation technology provides a simple and effective method to produce knockout animals.

The rat is an important animal model that has been used to understand human diseases^1^,^2^. Genetically modified (GM) rat strains are now being used to model human diseases in various research fields^3^,^4^. Engineered endonucleases, including zinc-finger nucleases (ZFNs), transcription activator-like effector nucleases (TALENs), and the clustered regularly interspaced short palindromic repeat (CRISPR)/CRISPR-associated (Cas) system, are invaluable tools for the rapid generation of GM animals including rats^5^,^6^,^7^,^8^. Importantly, these new technologies provide genome-editing approaches for a wide variety of organisms that were previously inaccessible without embryonic stem (ES) cells^9^,^10^,^11^ and induced pluripotent stem (iPS) cells^12^,^13^. GM animals are usually produced by microinjecting engineered endonucleases into pronuclear-stage embryos^5^,^6^. Although this method is now the gold standard, it requires sophisticated manual skills to prevent cell damage. Additionally, microinjection is not convenient when many cells need to be assessed simultaneously because the DNA/RNA has to be injected into embryos one by one using a micromanipulator. Electroporation is another method that introduces exogenous DNA/RNA into embryos. However, the current protocols require that the zona pellucida of the embryos is weakened by treatment with Tyrode's acid solution before electroporation for the efficient introduction of DNA^14^,^15^. To simplify these procedures, we introduced ZFN, TALEN, or CRISPR/Cas mRNA into intact rat embryos without weakening the zona pellucida by electroporation using the Super Electroporator NEPA 21 (NEPA GENE Co. Ltd., Chiba, Japan).

## Results

### Introduction of mRNA using an electroporator

**Figure 1:**
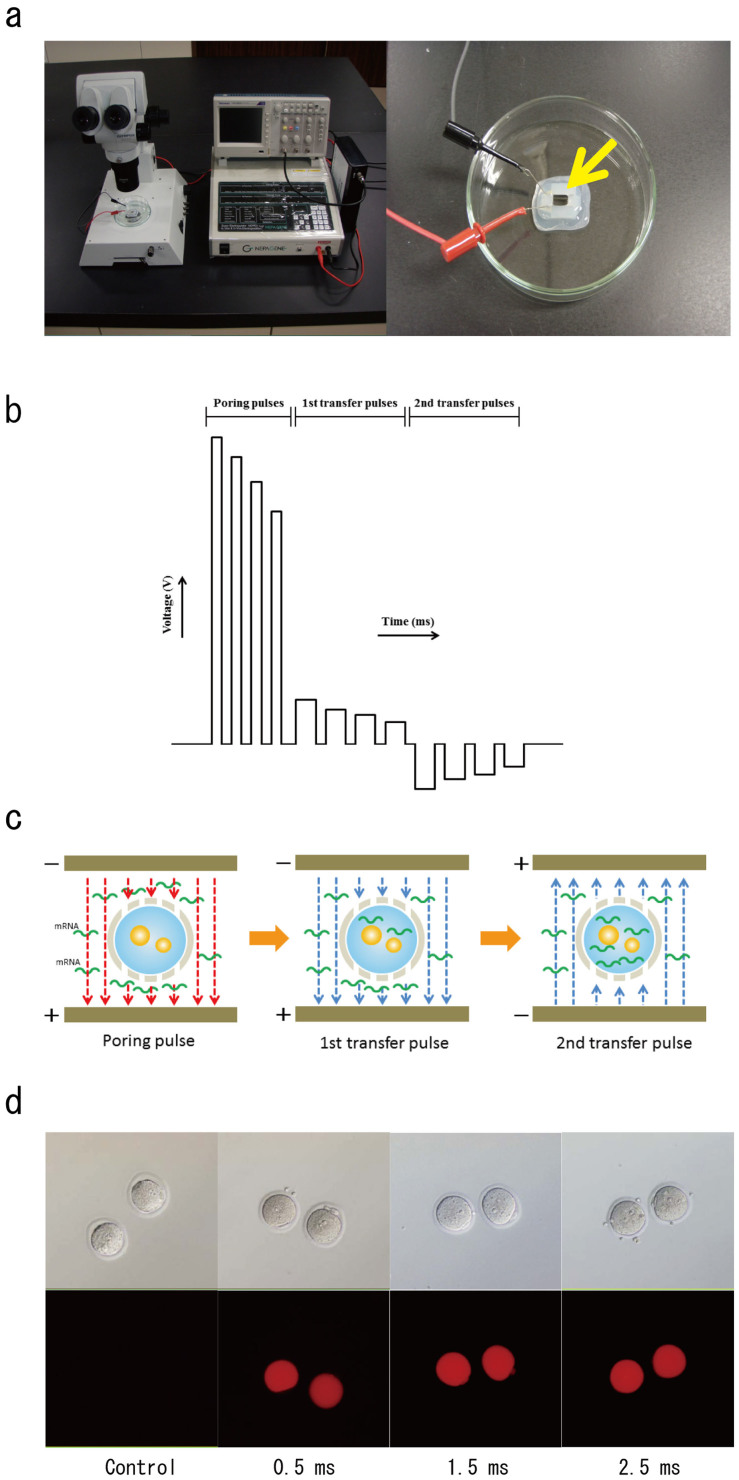
Introducing mRNA into embryos by electroporation. (a) Super Electroporator NEPA 21 (left) and glass chamber with metal plates (right). Embryos were placed in a line between the metal plates (arrow). (b) Illustration of electric pulses delivered by the electroporator. (c) The first poring pulse with high voltage and short duration makes micro-holes in the zona pellucida and oolemma (left), and mRNA in PBS was then moved into the cytoplasm with a few first transfer pulses with a low voltage and long duration after the poring pulse (middle). The mRNA was then transferred into oocytes by the polarity-changed second transfer pulse (right). (d) Fluorescence analysis of tetramethylrhodamine-labelled dextran that was introduced into embryos by electroporation with the pulse width adjusted to 0, 0.5, 1.5, and 2.5 ms.

ZFN, TALEN, or CRISPR mRNA was electroporated into embryos (Fig. 1a). Pronuclear-stage embryos were placed in a line on the glass chamber between metal plates that were filled with phosphate-buffered saline (PBS) containing mRNA (Fig. 1a). mRNAs were efficiently introduced into intact embryos with a 3-step pulse system (Fig. 1b). In the first step, the poring pulse makes micro-holes in the zona pellucida and oolemma. In the second step, several of the first transfer pulses transfer mRNA into the cytoplasm. In the third step, the polarity-changed second transfer pulse increases the chance of transferring mRNA into embryos (Fig. 1c).

### Introduction of tetramethylrhodamine-labelled dextran into intact pronuclear-stage embryos

To test the electroporation conditions to introduce materials into embryos, tetramethylrhodamine-labelled dextran (3 kDa molecular weight), which is easily visualised and nontoxic to living cells, was used. Pronuclear-stage embryos were collected from superovulated female rats the day after mating. Tetramethylrhodamine-labelled dextran was electroporated into intact rat embryos using the electroporator with the pulse width adjusted to 0, 0.5, 1.5, and 2.5 ms. Dextran was located in the whole cytoplasm of the embryos (Fig. 1d).

### Introduction of the ZFN or TALEN plasmid into fibroblast-like cells

ZFN and TALEN plasmids were designed to target exon 2 of the rat interleukin 2 receptor gamma (*Il2rg*) gene (Supplementary Fig. S1)^16^,^17^. To evaluate the activity of the ZFN and TALEN, we electroporated these expression vectors into rat fibroblast-like (Rat-1) cells. After 1 week, the cells were collected, and the genomic DNA was extracted and analysed for mutations using the Surveyor (Cel-1) nuclease assay. The Rat-1 cells that were transfected with the plasmids showed a cleavage activity of 2.6% for ZFN and 29.3% for TALEN compared with the negative control cells that were transfected with the GFP expression vector (Supplementary Fig. S2). Sequence analysis of the *Il2rg* locus also revealed mutation rates of 10.4% (10 mutations per 96 colonies screened) for ZFN and 19.8% (17 mutations per 86 colonies) for TALEN (Supplementary Fig. S3).

### Introduction of ZFN or TALEN mRNA into intact pronuclear-stage embryos

ZFN or TALEN mRNA was electroporated into intact rat pronuclear-stage embryos. The ZFN or TALEN mRNA was transcribed from the plasmids; mRNA concentrations of 40 μg/mL and 10 μg/mL were used for electroporation and microinjection, respectively. The transcribed mRNA was electroporated into intact embryos with a poring pulse (voltage: 225 V; pulse interval: 50 ms; pulse width: 0.5, 1.5, and 2.5 ms; number of pulses: 4). After electroporation, the embryos that developed to the 2-cell stage were transferred into the oviducts of pseudopregnant females. Of the embryos that were microinjected with ZFN mRNA, which served as a control in this study, 10% developed into offspring, and 33% of the offspring had an edited *Il2rg* locus (Table 1). In contrast, 31, 24, and 6% of the embryos that were electroporated with the pulse width adjusted to 0.5, 1.5, and 2.5 ms, respectively, developed into offspring. Of these offspring, 37, 73, and 75%, respectively, had an edited *Il2rg* locus (Table 1, Supplementary Fig. S4). The germline transmission of the ZFN electroporation-induced editing was also confirmed in the next generation (Supplementary Table S1).

Of the embryos that were microinjected with TALEN mRNA, 12% developed into offspring, and all 6 of the delivered pups had an edited *Il2rg* locus (Table 1). TALEN mRNA was electroporated into intact pronuclear-stage embryos with the same poring pulse that was used for the ZFN electroporation, and the pulse width was adjusted to 1.5 and 2.5 ms. Of the embryos that were electroporated with TALEN mRNA with the pulse width adjusted to 1.5 and 2.5 ms, 44 and 30%, respectively, developed into offspring, and 4 and 18%, respectively, had an edited *Il2rg* locus (Table 1, Supplementary Fig. S5).

### Introduction of *Cas9* mRNA and guide RNA into intact pronuclear-stage embryos

*Cas9* mRNA and single-stranded guide RNA (gRNA) targeting the rat *Il2rg* gene (Supplementary Fig. S1) were electroporated into intact rat pronuclear-stage embryos with a poring pulse (voltage: 225 V; pulse interval: 50 ms; pulse width: 1.5 and 2.5 ms; number of pulses: 4). *Cas9* mRNA and gRNA were used at concentrations of 2,000 and 1,000 μg/mL for electroporation and 100 and 50 μg/mL for microinjection, respectively. After the electroporation or microinjection, the embryos that developed to the 2-cell stage were transferred into the oviducts of pseudopregnant females. Of the embryos that were microinjected with *Cas9* mRNA and gRNA, which served as a control in this study, 41% developed into offspring, and 51% of the offspring had an edited *Il2rg* locus (Table 1, Supplementary Fig. S6). In contrast, 43 and 55% of the embryos that were electroporated with the pulse width adjusted to 1.5 and 2.5 ms, respectively, developed into offspring. Finally, 9% of the offspring derived from embryos that were electroporated with a pulse width adjusted to 2.5 ms had an edited *Il2rg* locus (Table 1, Supplementary Fig. S6).

## Discussion

Here, we established a method to produce knockout rats by introducing ZFN, TALEN, and CRISPR/Cas mRNA into intact embryos using electroporation. Introducing DNA/RNA into embryos by electroporation has been used to analyse the mechanism of embryo development and expression of DNA/RNA in preimplantation embryos *in vitro*. However, previous methods required that the zona pellucida was weakened with Tyrode's solution before electroporation^14^,^15^. The weakening of the zona pellucida may affect the subsequent embryonic development because of its importance in embryonic development *in vivo*^18^,^19^. We observed that tetramethylrhodamine-labelled dextran was present in the whole cytoplasm of the intact embryos after electroporation (Fig. 1d). Our method using the Super Electroporator NEPA 21 introduced satisfactory nucleases even if intact embryos were used. This method was simple and effective compared with the conventional electroporation methods that use embryos with a weakened or removed zona pellucida (Table 1).

In general, knockout animals have been produced by injecting targeted genes that were modified by homologous recombination into blastocysts of ES cells^11^. This process requires sophisticated manual skills, and the success rates of knockout animals varied in each strain^20^. Engineered endonucleases have been used to edit targeted genes in nematodes, flies, zebrafish, frogs, and human ES cells and iPS cells^21^,^22^. It was also reported that these mRNAs are highly active in rat embryos^5^,^6^,^16^,^23^. Engineered endonucleases make it possible to produce knockout animals even in strains and species that do not have established ES cell lines.

The electroporation method used in this study efficiently introduced ZFN, TALEN, and CRISPR mRNA into intact embryos with a 3-step pulse system (Fig. 1b). It was thought that this electroporation system could efficiently introduce mRNA into intact embryos by using two operating transfer pulses (Fig. 1c). The introduction of nucleases to embryos that have not been treated to weaken or remove the zona pellucida possibly reduces the time necessary for embryo manipulation, facilitating the healthy development of offspring after embryo transfer. In fact, electroporation with the 3-step pulse system drastically increased the survival rate of embryos that received ZFN (91% with the pulse width adjusted to 1.5 ms) compared with that from microinjection (44%) (Table 1). Importantly, the genome-editing rate was also significantly higher with the electroporation of ZFN (73%) than with microinjection (33%), indicating the efficient introduction of mRNA by the 3-step pulse system. In the case of TALEN and CRISPR, the survival rate was increased (98 and 91% with the pulse width adjusted to 2.5 ms, respectively), but the editing rate was not (18 and 9%) (Table 1). The size of the TALEN and *Cas9* mRNA, which is three times larger than that of the ZFN mRNA, might hamper its penetration through the zona pellucida. Although the editing rate of TALEN and CRISPR is sufficient for genome editing, further modifications of the electroporation conditions or an improved method that increases genome editing with the co-introduction of exonuclease 1 (*Exo1*) mRNA^24^ might be required. Further modifications should lead to increasing success rates of knockout animals.

In conclusion, we established a novel, efficient method for generating knockout rats that we called the “Technique for Animal Knockout system by Electroporation (TAKE)”. The electroporation protocol has high success rates without requiring special technical skills, and it is much faster than the microinjection of multiple cells. It is also applicable to embryos from various animal species. We believe that the TAKE method will facilitate the production of genetically engineered animals to be used to study gene functions and human diseases.

## Methods

### Animals

All animal care and procedures that were performed in this study conformed to the Guidelines for Animal Experiments of Kyoto University, and they were approved by the Animal Research Committee of Kyoto University. The F344/Stm rats used in this study were supplied by the National BioResource Project-Rat (Kyoto, Japan, http://www.anim.med.kyoto-u.ac.jp/NBR). Males that were older than 11 weeks and females that were 8 to 16 weeks old were used as sperm and oocyte donors, respectively. Jcl:Wistar females that were 8 to 16 weeks old were purchased from CLEA Japan Inc. (Tokyo, Japan) and were used as recipients for embryo transfer. All animals were maintained in an air-conditioned (temperature 24 ± 2°C, humidity 50 ± 10%) and light-controlled room (lights on from 07:00 to 19:00).

### Preparation of ZFN, TALEN, and CRISPR mRNA

Custom-designed ZFN plasmids from Sigma-Aldrich (St. Louis, MO, USA)^23^ and self-made TALEN and CRISPR plasmids were used. The protocol for TALEN assembly was previously described^17^. Plasmids expressing Cas9 (hCas9: ID#41815) and gRNA (gRNA_Cloning Vector: ID#41824) were obtained from Addgene (www.addgene.org/CRISPR). We designed the ZFN, TALEN, and CRISPR to target the rat *Il2rg* gene (Supplementary Fig. S1). ZFN, TALEN, and *Cas9* mRNA were transcribed *in vitro* using a MessageMax™ T7 mRNA transcription kit (Cambio, Cambridge, UK) and polyadenylated using an A-Plus™ Poly(A) polymerase tailing kit (Epicentre Biotechnologies, Madison, WI, USA). gRNA was transcribed with a MEGAshortscript T7 Kit (Life Technologies Co., Carlsbad, CA, USA). RNA was then purified using a MEGAClear™ kit (Life Technologies Co.). mRNA of ZFNs and TALENs was resuspended in PBS at 40 μg/mL for electroporation and 10 μg/mL for microinjection. *Cas9* mRNA and gRNA were resuspended in PBS at 2,000 and 1,000 μg/mL for electroporation and 100 and 50 μg/mL for microinjection, respectively.

### Cell culture and transfection

Rat-1 cells were obtained from the RIKEN BRC Cell Bank (Tsukuba, Japan, http://www.brc.riken.jp/lab/cell/english). The Rat-1 cells were cultured in Dulbecco's modified Eagle's medium (Life Technologies Co.) that was supplemented with 10% fetal bovine serum (Thermo Fisher Scientific Inc., Waltham, MA, USA) in a humidified atmosphere containing 5% CO_2_ at 37°C. The cells (1 × 10^6^) were suspended in 100 μL PBS, given 10 μg plasmid, and electroporated with the Super Electroporator NEPA 21 (NEPA GENE Co. Ltd., Chiba, Japan) under the following conditions: (A) pulse voltage, 275 V; pulse interval, 50 ms; pulse width, 2.5 ms; and pulse number, 2; or (B) pulse voltage, 275 V; pulse interval, 50 ms; pulse width, 1.0 ms; and pulse number, 3. The *in vitro* transfer experiment was replicated four times.

### Collection of embryos

Females were induced to superovulate by an intraperitoneal injection of 150 IU/kg pregnant mare serum gonadotropin (ASKA Pharmaceutical Co. Ltd., Tokyo, Japan) followed by an injection of 75 IU/kg human chorionic gonadotropin (ASKA Pharmaceutical Co. Ltd.) 48 h later. Females were then mated with males of the same strain overnight. Pronuclear-stage embryos were collected from oviducts of females the day after mating, and they were kept in modified Krebs-Ringer bicarbonate (mKRB)^25^ before electroporation.

### Introduction of ZFN, TALEN, and CRISPR mRNA into intact embryos by electroporation

Tetramethylrhodamine-labelled dextran (3 kDa; Life Technologies Co.) that was diluted in PBS to a concentration of 2 mg/mL was introduced to observe the localisation of external material in the embryos after electroporation. Pronuclear-stage embryos were placed in a line on the glass chamber between metal plates that were filled with PBS containing tetramethylrhodamine-labelled dextran (Fig. 1a). The poring pulse (voltage: 225 V, pulse interval: 50 ms, number of pulses: 4) was selected, and the pulse width was adjusted to 0.5, 1.5, and 2.5 ms. The localisation of tetramethylrhodamine-labelled dextran was observed in all embryos in each group using an inverted microscope with fluorescence (Olympus Co., Tokyo, Japan). Embryos that were exposed to PBS with tetramethylrhodamine-labelled dextran without electroporation were used as a control (Fig. 1d).

ZFN, TALEN, or CRISPR mRNA was introduced into embryos using the same method as that used to introduce tetramethylrhodamine-labelled dextran. Pronuclear-stage embryos were electroporated with the same poring pulse that was used for the tetramethylrhodamine-labelled dextran, and different pulse widths (0.5, 1.5, and 2.5 ms) were used for the PBS containing ZFN mRNA. TALEN or *Cas9* mRNA and gRNA were electroporated into embryos with the same poring pulse and different pulse widths (1.5 and 2.5 ms). Embryos that received microinjected mRNA in the pronucleus were used as controls. All embryos in each group were cultured in mKRB medium at 37°C under 5% CO_2_ and 95% air.

### Embryo transfer

Embryos that developed to the 2-cell stage after the introduction of ZFN, TALEN, or CRISPR mRNA were transferred into the oviducts of surrogate females that were mated with vasectomised males the day before the embryo transfer. The number of offspring that were born naturally was counted at 21 days of gestation.

### Analysis of gene editing in offspring

Editing of the targeted gene was analysed using genomic DNA that was extracted from blood that was adhered to FTA cards using a GENEXTRACTOR TA-100 automatic DNA purification system (Takara Bio Inc., Shiga, Japan). PCR was performed in a total volume of 15 μL under the following conditions: 1 cycle of 94°C for 3 min; 35 cycles of 94°C for 30 s, 60°C for 1 min, and 72°C for 45 s; and 1 cycle of 72°C for 3 min. The final reaction mixture contained 100 ng genomic DNA, 200 μM dNTPs, 1.0 mM MgCl_2_, 0.66 μM each primer, and 0.4 U Taq DNA polymerase (Life Technologies Co.). To edit the cleavage site in the genome at the *Il2rg* locus, two primer sets were designed to amplify small (292 bp) and large (3,158 bp) fragments as shown in Supplementary Fig. S1. The PCR products were directly sequenced using the BigDye terminator v3.1 cycle sequencing mix and the standard protocol for an Applied Biosystems 3130 DNA Sequencer (Life Technologies Co.).

### Analysis of germ-line transmission

Some offspring with deletions of the targeted gene that were derived from embryos electroporated with ZFN mRNA using a pulse width of 1.5 ms were selected. They mated naturally with wildtype males or females after they had matured. We analysed the edited gene in the offspring of the next generation.

## Author Contributions

T.K. designed the work and performed the experiments. T.S. and T.Y. constructed the TALEN plasmid. T.K. and T.M. wrote the manuscript. All authors reviewed the manuscript before submission.

## Supplementary Material

Supplementary Information Supplementary information

## Figures and Tables

**Table 1:** *In vivo* development of embryos with ZFN, TALEN, or CRISPR mRNA that was introduced by microinjection or electroporation

mRNA	Methods	Pulse width (ms)	No. of embryos examined	No. of 2-cell embryos (%)^a^	No. of offspring (%)^a^	No. of offspring with mutation (%)^b^	% (no. of offspring with mutation/no. of embryos examined)
ZFN	Microinjection	–	93	41 (44)	9 (10)	3 (33)	3.2
	Electroporation	0.5	61	58 (95)***	19 (31)**	7 (37)	11.5
		1.5	63	57 (91)***	15 (24)*	11 (73)	17.5**
		2.5	66	16 (24)*	4 (6)	3 (75)	4.5
TALEN	Microinjection^c^	–	52	20 (39)	6 (12)	6 (100)	11.5
	Electroporation	1.5	57	55 (97)***	25 (44)***	1 (4)	1.8
		2.5	57	56 (98)***	17 (30)*	3 (18)	5.3
CRISPR	Microinjection	–	120	100 (83)	41 (41)	21 (51)	17.5
	Electroporation	1.5	64	58 (91)	25 (43)	0 (0)	0***
		2.5	64	58 (91)	32 (55)*	3 (9)	4.7*

^a^Calculated from the number of embryos examined.

^b^Calculated from the number of offspring.

^c^Results from reference 17.

*P < 0.05,

**P < 0.01,

***P < 0.001 vs microinjection by Fisher's exact test.
