# Supplementary material for: Simple knockout by electroporation of engineered endonucleases into intact rat embryos
Source: Sci Rep. 2014 Oct 1;4:6382. doi: 10.1038/srep06382 (PMC4180828; doi:10.1038/srep06382)
Supplement: Supplementary Information [file srep06382-s1.pdf]

Supplementary Information

Simple knockout by electroporation of engineered endonucleases into intact rat embryos

Takehito Kaneko, Tetsushi Sakuma, Takashi Yamamoto, Tomoji Mashimo

Supplementary Table S1. Germ-line transmission in offspring that were derived from embryos with *Il2rg*-targeted ZFN mRNA.

		Offspring		Offspring with an edited target gene	
Fathers	Mothers	Males	Females	Males	Females
G0 Δ13/Y	+/+	8	5	0	5
G0 Δ7/Y	+/+	7	5	0	5
G0 Δ5/Y	+/+	6	4	0	2
+/Y	F1 Δ13/+	5	6	3	3
+/Y	F1 Δ13/+	9	1	5	1

Supplementary Figure Legends

Supplementary Figure S1. ZFN and TALEN pairs and gRNA designed to target the *Il2rg* locus and primer sequences used for the PCR analysis of the *Il2rg* gene. Each exon is underlined. The start codon is indicated by a red box. The two primer sets (small and large) for the PCR analysis of *Il2rg* are shown by boxes.

Supplementary Figure S2. Validation of ZFN and TALEN activity in rat fibroblasts (Rat-1). (a) ZFN or TALEN expression vectors, and the GFP expression vector as a negative control, were electroporated into Rat-1 fibroblast cells using the Super Electroporator NEPA 21. The electroporation conditions are as follows: (A) pulse voltage, 275 V; pulse interval, 50 ms; pulse width, 2.5 ms; and pulse number, 2; or (B) pulse voltage, 275 V; pulse interval, 50 ms; pulse width, 1.0 ms; and pulse number, 3. The Surveyor (Cel-I) nuclease assay was performed to detect mutations in the *Il2rg* locus. (b) The Surveyor assay indicated the activity of ZFN and TALEN in Rat-1 cells. Data are expressed as means ± SEM (n = 4).

Supplementary Figure S3. Sequencing assay for ZFN- and TALEN-induced mutations in the *Il2rg*-targeted locus in rat fibroblasts (Rat-1). Multiple deletions or insertions, which are depicted using yellow dashes or letters, respectively, are aligned along the wildtype sequence

that is shown on the top line.

Supplementary Figure S4. Sequencing assay for ZFN-induced mutations in the *Il2rg*-targeted locus in the edited offspring. Multiple deletions or insertions, which are depicted using yellow dashes or letters, respectively, are aligned along the wildtype sequence that is shown on the top line. MI, microinjection; EP, electroporation.

Supplementary Figure S5. Sequencing assay for TALEN-induced mutations in the *Il2rg*-targeted locus in the edited offspring. Multiple deletions, which are depicted using yellow dashes, are aligned along the wildtype sequence that is shown on the top line. MI, microinjection; EP, electroporation.

Supplementary Figure S6. Sequencing assay for CRISPR/Cas-induced mutations in the *Il2rg*-targeted locus in the edited offspring. Multiple mutations, which are depicted using yellow, are aligned along the wildtype sequence that is shown on the top line. MI, microinjection; EP, electroporation.

Primer Il2rg_Large_F
AAATCAGGCAGAGGCAAAGAGCAGCTGGCTGGTTCCTACCTTTGTTTACCTGTGTTTTGGAGAATCTCCACAT
CTATGCTGTAGAACTCATTACAGTACATTGTATTATTTATATGTTCCCCACTTATCTCTGAGCTTCTAAAATG
ATGATGTCTTATTTGTCTTATGTTCTCAGAACATAAGCACTGTACCCAGCACATATTAAAGACTCAATAAATG
TTGGCTGGATAAACAATTTCAGTAAATGGCTTCTCCAATCAACCCTGTGCTCTGAGGGAAGGTAAATCTAGCC
ACAGAATGAAGAATGGACGGGAGAGCAGAGGCCCTTGAGAAAGGGGACCAGTTTGTGGGTTACGGGAATAATC
ATGACTGGAGGTAATGAAAGGCTGATTTAGCACAGTGGCTGCGGTTAACAGAAAGGAGGAAACTACTGGGAGA
AATACCGCAGAAACAGGGCTGATTGGATTCTCGGTGTGAGAGAGAAGGGAGGGACATGAAGAGGATCCTGAGG
TTTCAAGTGTGGCAGGTGATGATGCTATTAAGCAGGACAAGTAAGACAGAAAGAGAAGCAGATTTGCAGGGAG
ATAAGGAGCCTGAAGTTAGTACTGTTGTCCCTCCATCTTAGACGCCAGCTCTCACCAGGGACAACAGGGACCA
AACTCAGGCAGCAGTTGGGGGTGGTTATTCTAGTTTGATTAGGTCAGAGGAAAGACAGCTGTGTGTCTACCCG
CATGAATCAAGTCAGTATTTTCCATCTATCCTTCCTAGACTGTACAACTTTGACAGAGGTTTAAGATAGCCTA
GAGGGAAAAGGTGGTTGGGAATGAAGGTGTGTGGTGGGGGTGGGTGTTCAGCAGAGTCTTTCTGGACCTAGGT
GGCCTGAAGAGCCTTAATTCTGTGACACTGTTGTTGATCAAGTATTAATCTTGACAGAACATCACCTTAGAGC
AGAACCCAAATCTCCCAGGGGACTTAGCTTATGTCACTGAACACATTTACTAACCCCCCTCCCTCTACAGCGT
GGTTTCTAAGGTTCTTTCCACCGGAAACTACGACAAAAGGAAATGTATGGGTGGGGAGGGCTTGTGGGAGAGT
GGTTCAGGGTTCTGACACAGACTACACCCAGAGAAAGAAGAGCAAGCGCCATGTTGAAACCATTATTGCCATC
Exon 1
TAGATCCTTCTTACTCCTTCAGCTGCTTCTGCTGAGGGTAGGGTGGAGCTCCAAGGTCCTCATGTCCAGTGGG
AATGAAGACACCAAATCTGGTAGGAAATCTAAGACCAGAGGGGATTGCTGAGAGGAAGGCTATGGGGAAAGGG
CTGTATAGAAGCTCTTTCTATACAATCTGGAGCCTGCTAGTGCTCACTATAATGACTAAAAAGAAGTGTGGGG
AAGGGGAGGGGAGGGGAGGGCTGTCTGTCTCACTACTGCTTCTTCTGACCAAGATTTCTTTTTCTTCCACTCC
Primer Il2rg_Small_F
ACTGTTTCATTTTCTTCCCAAACTTAGATCTCTTGCTGACTTCTATGGACCTTAAACATCTCAGTGTTCCTAC

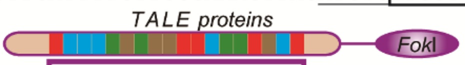

TCTGCCCCTCCCAGAGGTTCAATGCTTTGTGTTCAATGTCGAGTATATGAATTGCACTTGGAATAGCAGTTCT
Exon 2

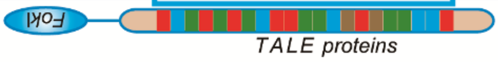

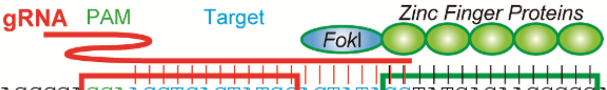

GAGCCTCAGCCGACCAACCTCACTATGCACTATAGGTATGAGAAGGGGGAGGGGTAGTACAGGAAGAAGAGAA

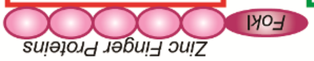

GGTGGGTTAGCTGAGAGAGACGGGGGAGCAAAAAAGTGGGTAGCCAGCTCCTCAGGTACCATACCAGTTTCTC
ATGGGATAAGTTATCAGTTCAGACCAGATGAAGCTAGGCTATGGGCAGATGTGGTACCTACCTATGTTTGGCC
Primer Il2rg_Small_R
Exon 3
CATCATTCTTTTGCCTTGTAACCCTTCTCTAGGTACAAGGGATCTGATAATAATACATTCCAGGAGTGCAGCC
ACTATCTGTTCTCAAAAGAGATTACTTCTGGCTGTCAGATACAAAAAGAAGATATCCAGCTCTACCAGACATT
TGTTGTCCAGCTTCAGGACCCCCAGAAACCCCAGAGGCGAGCCGAACAGAAGCTAAACCTACAGAATCTTGGT
AATCGGGAAAGAAGTGGCCAAGAGGCCAGGGAGCTTAAAGGCACTGGAGTTTATAGATTGTTCTTTTCTCATT
GTTGGTCATGGGCAGAAAGGCGAAGATGGGGGGGGGCGGGGAGGGATGAAGGGAATTACCTCCAAGATCCTG
ACTTGTCTAGGCCAGGGCAATGACCACGCACACACATATTCCAGTGATCCCATGGGCTCCAGAGAATCTAACA
CTTTATAACCTGAGTGAATCTCAGGTAGAACTGAGGTGGAAAAGCAGATACATAGAACGCTGTTTACAATACT
TGGTGCAGTACCGGAGCAACCGAGATCGAAGCTGGACGGTGAGTGACTTGGCATTGTGGATCAGTGGCTAAGG
Exon 4
CCAAGTAAGAGGAGAAAGGGTTCAAGTAGCCAGACAGAAAAACCTAACACCAGGTCCCAGTTCTCTGCCTCCC
AGCCCCTCTGTCCATACTTTCACTTTTTCTTTCTGACATCATCAGTGAGCTTTCCCTTTGTCTCTCCCACCAT
CCTCTTCCTGATGACAGACATAAAGGAAGCCAGCGAGTGGGTCTGGGGGGAATTAAGTTAGAGAAGGGGAGCA
GTATGTTCTCATTAGTCAGGAGAGGAGCAAAGTAGAACAGGGAGGAACCAGAACTGGTTTTTTGTTTGTTTTC
CTGGTACTGGAAAGACCAAGAAATTAGAAGAATCAGAAGATGACATTGAGGTATAGGCAAGAATGTTCCCATT
CTCTCTTCCTTTATAGATAACCTCTTTCCTTCGCCCTTCTTCTCCCTAGGAACAAATAGTGGATCATGAGCCT
Exon 5
AGATTCTCCCTGCCTAGTGTGGATGAACAGAAGCTGTACACATTTCGGGTTCGGAGCCGCTTTAACCCGATCT
GTGGAAGTACTCAACAGTGGAGTAAATGGAGCCAACCAATCCACTGGGGGAGCCATACTGCAGAGGGTAAAGT
GACCCAAATGCATCATCCCTAAATCATTCACCCATACCCTAACCTTTGGAACACACTGTCACTACCATTTGCT
TTATCTCTCTAGCCCTAAGTCTCAGCCCCCTTACTGTTTAGCCTCAATCACTATGAAGTAGGGTTTTTTCTAT
GTAGGGATGAGAAGGGGTG
Primer Il2rg_Large_R

Supplementary Figure S1

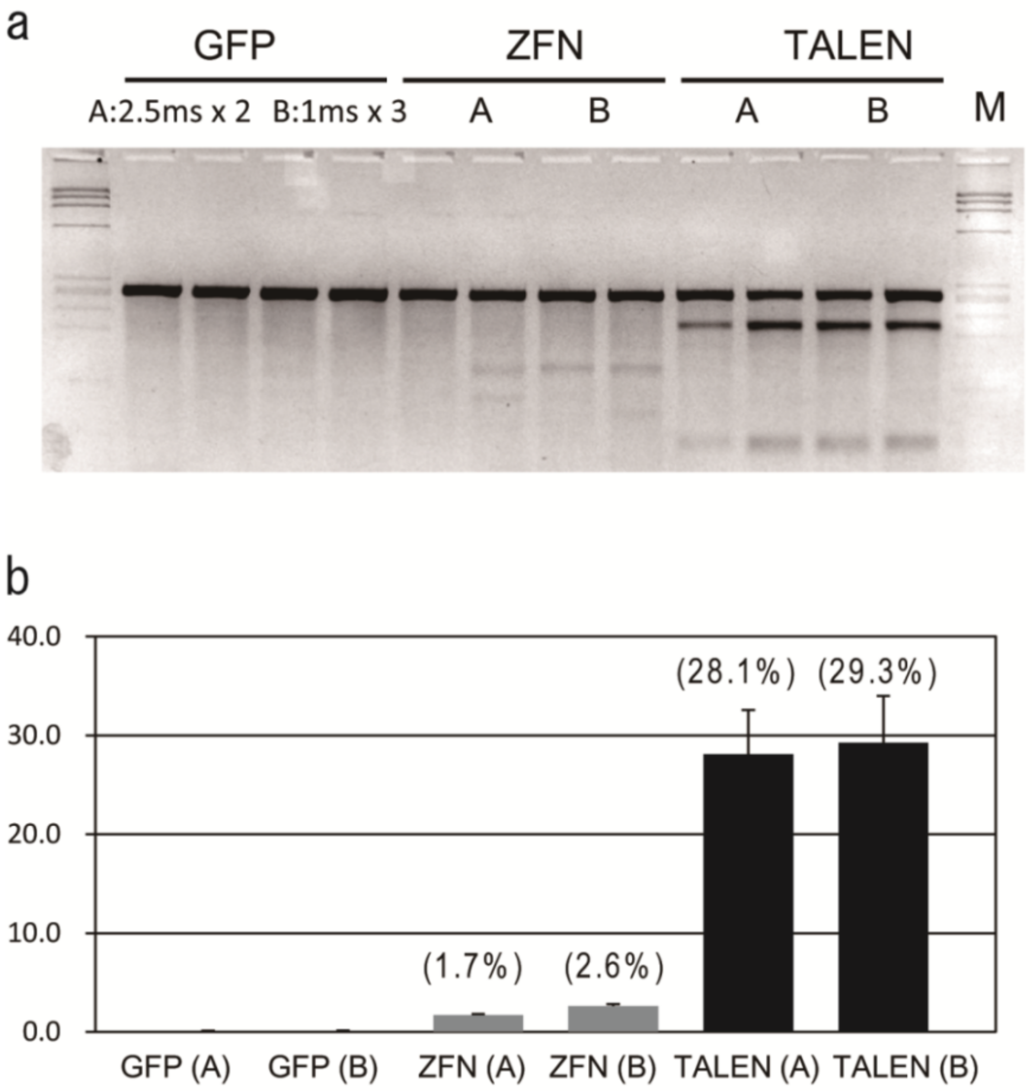

Supplementary Figure S2

ZFN-induced mutations: 10/96 colony (10.4%)

```
Wild-type  GCCGACCAACCTCACTATGCACTATAGGTATGAGAAGGGGGAGGGG
       #6  GCCGACCAACCTCACTATGCACTATTATAGGTATGAGAAGGGGGAGGGG
      #10  GCCGACCAACCTCACTATGCACTATCTATAGGTATGAGAAGGGGGAGGGG
      #60  CAGTT--------------(Δ83bp)---------------AGCTG
      #24  GCCGACCAACCTCACTATGCACTATGGGTATGAGAAGGGGGAGGGG
```

TALEN-induced mutations: 17/86 colony (19.8%)

```
Wild-type  GCCCCTCCCAGAGGTTCAATGCTTTGTGTTCAATGTCGAGTATATGAATTGCACTTGGAATAGC
       #3  GCCCCTCCCAGAGGTTCAATGCTTTGTGTTCAATGTCGAGTAG----(Δ163bp)----ACCAG
       #5  GCCCCTCCCAGAGGTTCAATGCTTTGTGTTCA---TCGAGTATATGAATTGCACTTGGAATAGC
       #9  GCCCCTCCCAGAGGTTCAATGCTTTGTGTT---------GTATATGAATTGCACTTGGAATAGC
      #12  GCCCCTCCCAGAGGTTCAATGCTTTGTGT-------CGAGTATATGAATTGCACTTGGAATAGC
      #17  GCCCCTCCCAGAGGTTCAATGCTTT--------TGTCGAGTATATGAATTGCACTTGGAATAGC
      #52  GCCCCTCCCAGAGGTTCAATGCTTTGTGTTCAATGTTGAGTATATGAATTGCACTTGGAATAGC
```

Supplementary Figure S3

```
F344/Stm  GCCGACCAACCTCACTATGCACTATAGGTATGAGAAGGGGGAGGGG

MI        GCCGACCAACCTCAC---(Δ13bp)---TATGAGAAGGGGGAGGGG
          GCCGACCAACCTCACTATGCA---TA-GTATGAGAAGGGGGAGGGG

EP        GCCGACCAACCTCAC---(Δ13bp)---TATGAGAAGGGGGAGGGG
(0.5ms)   GCCGACCAACCTCACTATGC-----AGGTATGAGAAGGGGGAGGGG
          GCCGACCAACCTCACTATGCA-------TATGAGAAGGGGGAGGGG
          GGAGG-------------(Δ262bp)---------------AAGAA
          GCCGACCAACCTCACTATGCA---TAGGTATGAGAAGGGGGAGGGG
          GCCGACCAACCTCACTATG------AGGTATGAGAAGGGGGAGGGG
          GCCGACCAACCTCACTATGCATGAGAGGTATGAGAAGGGGGAGGGG

EP        GCCGACCAACCTCA---(Δ12bp)--GGTATGAGAAGGGGGAGGGG
(1.5ms)   GCCGACCAACCTCACTATGCATTTACTAACATACAGGTGGTAG
                                  TAGGTATGAGAAGGGGGAGGGG
          GGAGGCCAACCTCACTATGCAC-----GTATGAGAAGGGGGAGGGG
          GCCAT-----------(Δ494bp)---GTATGAGAAGGGGGAGGGG
          GCCGACCAACCTCACTATG--(Δ9bp)-TATGAGAAGGGGGAGGGG
          CAAGG-------------(Δ827bp)---------------AAAGA
          GCCGACCAACCTCACTATGCAC--TAGGTATGAGAAGGGGGAGGGG
          AGAAG--------(Δ261bp)------GTATGAGAAGGGGGAGGGG
          CTGAG--------(Δ33bp)--------TATGAGAAGGGGGAGGGG
          GCCGACCAACCTCACTATGCA---TAGGTATGAGAAGGGGGAGGGG

EP        GCCGACCAACCTCACTATGCACTATTTAGGTATGAGAAGGGGGAGGGG
(2.5ms)   GCCGACCAACCTCACTATGCATACACAAAGCATA----GGGAGGGG
          GCCGACCAACCTCACTATGCA-----GGTATGAGAAGGGGGAGGGG
          GCCGACCAACCTCACTATGCATACACAAAGCATATGAGAAGGGGGAGGGG
          CTTTG-------------(Δ650bp)---------------ACCAC
```

Supplementary Figure S4

F344/Stm	GCCCCTCCCAGAGGTTCAATGCTTTGTGTTCAATGTCGAGTATATGAATTGCACTTGGAATAGC
MI	GCCCCTCCCAGAGGTTCAATGCTTTGT-------GTCGAGTATATGAATTGCACTTGGAATAGC
	AGGAA-----------------------(Δ286bp)--------GAATTGCACTTGGAATAGC
	GCCCCTCCCAGAGGTTCAATGCTTTGT---------CGAGTATATGAATTGCACTTGGAATAGC
EP (1.5ms)	GCCCCTCCCAGAGGTTCAATGCTTTGTGTTC-ATGTCGAGTATATGAATTGCACTTGGAATAGC
EP (2.5ms)	GCCCCTCCCAGAGGTTCAATGCTTTGTGTTC-ATGTCGAGTATATGAATTGCACTTGGAATAGC

Supplementary Figure S5

```
F344/Stm  AGCCTCAGCCGACCAACCTCACTATGCACTATAGGTATGAGAA

MI        AGCCTCAGCCGACCAACCTTTTTAAAAAGCTCTATGCACTATAGGTATGAGAA
          AGCCTCAGCCGACCAACCTTCACTATGCACTATAGGTATGAGAA
          AGCCTCAGCCGACCAACCATCACTATGCACTATAGGTATGAGAA
          AGCCTCAGCCGACCAACC---GTATGCACTATAGGTATGAGAA
          AGCCTCAGCCGACCAAC----CTATGCACTATAGGTATGAGAA
          AGCCTCAGCCGACCA-----ACTATGCACTATAGGTATGAGAA
          AGCCTCAGCCGACC--------TATGCACTATAGGTATGAGAA
          AGCCTCAGCCGACCA--------ATGCACTATAGGTATGAGAA
          AGCCTCAGCCGAC-------------CACTATAGGTATGAGAA
          AGCCTCAG--------------TATGCACTATAGGTATGAGAA
          AGCCTCAGCCGACCAACCT-------------AGGT-TGAGAA
          GAATA----(Δ29bp)----ACTATGCACTATAGGTATGAGAA
          CTGAG-------------(Δ85bp)-------------AGAGA
          AGCCTCAGCCGACCAA--------(Δ87bp)-------AAAAG

EP        AGCCTCAGCCGACCAACCTTTCACTATGCACTATAGGTATGAGAA
(2.5ms)   AGCCTCAGCCGACCAACCT---TATGCACTATAGGTATGAGAA
          AGCCTCAGCCGACCA---------TACTCTATAGTTATGAGAA
```

Supplementary Figure S6
